# Assessment of the thoracic aorta after aortic root replacement and/or ascending aortic surgery using 3D relaxation-enhanced angiography without contrast and triggering

**DOI:** 10.3389/fcvm.2025.1532661

**Published:** 2025-03-12

**Authors:** Carsten Gietzen, Jan Paul Janssen, Juliana Tristram, Burak Cagman, Kenan Kaya, Robert Terzis, Roman Gertz, Thorsten Gietzen, Henry Pennig, Alexander C. Bunck, David Maintz, Thorsten Persigehl, Navid Mader, Kilian Weiss, Lenhard Pennig

**Affiliations:** ^1^ Institute for Diagnostic and Interventional Radiology, Faculty of Medicine and University Hospital Cologne, University of Cologne, Cologne, Germany; ^2^ Department of Cardiac Surgery, Heart Center, Faculty of Medicine and University Hospital Cologne, University of Cologne, Cologne, Germany; ^3^ Department of Cardiology, Heart Center, Faculty of Medicine and University Hospital Cologne, University of Cologne, Cologne, Germany; ^4^ Department for Orthopedic and Trauma Surgery, University Hospital of Bonn, Bonn, Germany; ^5^ Philips Healthcare Germany, Hamburg, Germany

**Keywords:** ascending aorta, magnetic resonance angiography, non-contrast-enhanced magnetic resonance angiography, connective tissue diseases, aortic surgery

## Abstract

**Objective:**

Relaxation-Enhanced Angiography without Contrast and Triggering (REACT) is a novel 3D isotropic flow-independent non-contrast-enhanced MRA (non-CE-MRA) and has shown promising results in imaging of the thoracic aorta, primarily in patients without prior aortic surgery. The purpose of this study was to evaluate the performance of REACT after surgery of the aortic root and/or ascending aorta by performing an intraindividual comparison to CE-MRA.

**Material and methods:**

This retrospective single center study included 58 MRI studies of 34 patients [mean age at first examination 45.64 ± 11.13 years, 31 (53.44%) female] after ascending aortic surgery. MRI was performed at 1.5T using REACT (ECG- and respiratory-triggering, Compressed SENSE factor 9, acquired spatial resolution 1.69 × 1.70 × 1.70 mm^3^) and untriggered 3D CE-MRA. Independently, two radiologists measured maximum and minimum vessel diameters (inner-edge) and evaluated image quality and motion artifacts on 5-point scales (5 = excellent) for the following levels: mid-graft, distal anastomosis, ascending aorta, aortic arch, and descending aorta. Additionally, readers evaluated MRAs for the presence of aortic dissection (AD) and graded the quality of depiction as well as their diagnostic confidence using 5-point scales (5 = excellent).

**Results:**

Vessel diameters were comparable between CE-MRA and REACT (total acquisition time: 05:42 ± 00:38 min) with good to excellent intersequence agreement (ICC = 0.86–0.96). At the distal anastomosis (minimum/maximum, *p* < .001/*p* = .002) and at the ascending aorta (minimum/maximum, *p* = .002/*p* = .06), CE-MRA yielded slightly larger diameters. Image quality for all levels combined was higher in REACT [median (IQR); 3.6 (3.2–3.93) vs. 3.9 (3.6–4.13), *p* = .002], with statistically significant differences at mid-graft [3.0 (2.5–3.63) vs. 4.0 (4.0–4.0), *p* < .001] and ascending aorta [3.25 (3.0–4.0) vs. 4.0 (3.5–4.0), *p* < .001]. Motion artifacts were more present in CE-MRA at all levels (*p* < .001). Using CE-MRA as the standard of reference, readers detected all 25 cases of residual AD [Stanford type A: 21 (84.0%); Stanford type B: 4 (16.0%)] in REACT with equal quality of depiction [4.0 (3.0–4.5) vs. 4.0 (3.0–4.0), *p* = .41] and diagnostic confidence [4.0 (3.0–4.0) vs. 4.0 (3.0–4.0), *p* = .81) in both sequences.

**Conclusions:**

This study indicates the feasibility of REACT for assessment of the thoracic aorta after ascending aortic surgery and expands its clinical use for gadolinium-free MRA to these patients.

## Introduction

In recent years, cardiovascular surgery societies have observed a notable increase in the annually number of aortic procedures (1). Given this epidemiological trend and the challenges of aortic surgery with a high risk of perioperative mortality and morbidity (2), the aorta has to be regarded as an independent organ with distinct imaging and treatment strategies to enhance patient outcomes (3). Aortic dilatation represents a common underlying aortic disease, affecting 5–10 per 100.000 individuals per year (4) and potentially leads to complications, e.g., aneurysm, dissection (AD), and rupture (5). Bentall procedure, referring to composite graft replacement of the aortic valve, root, and ascending aorta with direct reimplantation of the coronary arteries into the graft, is considered the preferred surgical approach in aortic root aneurysms involving a structurally diseased aortic valve as well as in AD (6). Nevertheless, the risk of postoperative bleeding and delayed adverse effects, is contingent upon the specific type of valve prosthesis utilized (mechanical vs. biological), remain to be considered (7). In contrast, valve-sparing surgical techniques, e.g., David operation, have demonstrated lower in-hospital mortality rates, due to a reduction in the incidence of valve-related complications and a lower bleeding risk (8). When aortic valve and root anatomy are suitable (9), consequently David operation, despite its complexity, is preferable in elective aortic root replacement (10). In accordance with the current guidelines (11), imaging surveillance utilizing computed tomography angiography (CTA), magnetic resonance angiography (MRA) and transthoracic echocardiography (TTE) is recommended at 1, 6 and 12 months postoperatively, with subsequent annual monitoring to identify potential post-surgical complications, including progressive growth of the native aorta, dissection or anastomotic complications (11–15). Of note, the choice of imaging modality and time interval between examinations vary between patients according to their risk, being defined by the location of aortic disease, type of treatment, and underlying aortic pathology (16). Furthermore, follow-up of acute aortic syndrome patients is characterized by a higher rate of complications and a higher need for reoperation, compared to follow-up for elective surgery in unruptured aortic aneurysms (11). Due to these recommendations, repetitive aortic imaging after aortic surgery is mandatory and leads to a high burden of ionizing radiation and iodinated contrast agents, when CTA is employed (13). On the contrary, contrast-enhanced MRA (CE-MRA) represents a radiation-free method for follow-up imaging after aortic surgery (17, 18). Nevertheless, CE-MRA is not without inherent limitations, including the costs for gadolinium-based contrast agents, the time required for patient preparation, and the potential for technical failure due to mistiming between bolus application and data acquisition (19). Moreover, the potential adverse effects of contrast agents, including allergic reactions (20) and uncertain effects of the long-term retention of gadolinium (21), have prompted the development of non-CE-MRA techniques (22).

2D/3D balanced SSFP (bSSFP) is the most often used sequence for the depiction of thoracoabdominal vessels (23, 24). Recently, a novel flow-independent non-CE-MRA technique, named Relaxation-Enhanced Angiography without Contrast and Triggering (REACT), has been developed and allows for the acquisition of 3D isotropic non-CE-MRA over a wide field of view (25). REACT has already demonstrated encouraging results in imaging of the supraaortal arteries (26–28), the pulmonary vasculature (29), congenital heart disease (30, 31), and the thoracic aorta (32, 33). However, previous studies primarily included patients without history of prior aortic surgery (32, 33). Consequently, the performance of REACT after aortic surgery with inherent challenges, e.g., artifacts due to grafts or other surgical material (34), is unknown.

The purpose of this study was to evaluate the performance of REACT in patients after aortic root replacement and/or surgery of the ascending aorta, by performing an intraindividual comparison of aortic diameters, subjective image quality, artifacts, and assessment of AD to CE-MRA.

## Materials and methods

The institutional review board approved this single-center study (reference number: 23–1,167-retro). Given its retrospective design, the requirement for written informed consent from the patient cohort was waived.

### Study population

**Figure 1:**
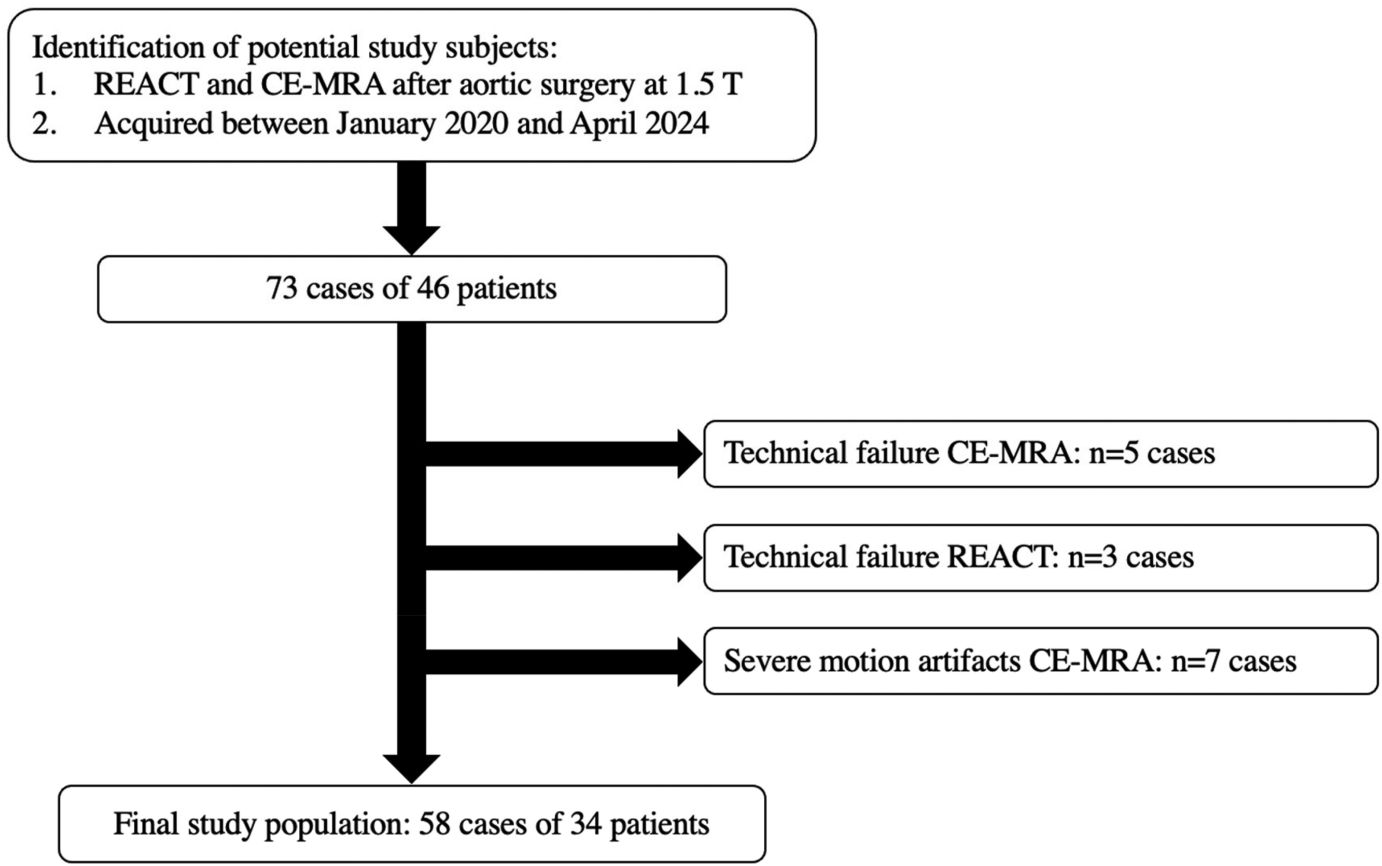
Workflow for inclusion and exclusion of cases. CE-MRA, contrast-enhanced magnetic resonance angiography; REACT, relaxation-enhanced angiography without contrast and triggering; T, tesla; *n*, numbers.

The authors conducted a review of the institutional image database at a tertiary care university hospital for aortic MRI examinations after aortic root replacement and/or ascending aortic surgery performed between January 2020 and April 2024. The study included patients, who had undergone a complete thoracic aorta MRI protocol at 1.5T, which included both REACT and CE-MRA of the thoracic aorta. Patients were excluded if they exhibited severe motion artifacts or technical failure in any MRA sequence, as assessed by a board-certified cardiovascular radiologist with eight years of experience in cardiovascular MRI (L.P.). Since some patients underwent repetitive imaging of the aorta after surgery, they are referred to as cases in this study. Overall, 73 cases of 46 patients with dual MRA imaging after aortic surgery were identified. Seven cases were excluded due to motion artifacts in CE-MRA. Five cases were excluded due to technical failure in CE-MRA, 3 in REACT. Consequently, the final study population consisted of 58 cases in 34 patients. The workflow for inclusion and exclusion of patients is depicted in Figure 1.

The following data were obtained from the medical charts or observed during examinations: Patient age, gender, body mass index (BMI), cardiovascular risk factors, underlying aortic disease [diagnosis of Marfan syndrome (MFS) was given based on a (likely) pathogenic fibrillin-1 gene variant], surgical procedure, previous history of cardiac surgery and acquisition time of MRAs including time needed for image reconstruction.

### MRI

Magnetic resonance imaging (MRI) was conducted using a commercially available whole body 1.5T MRI system (Philips Ingenia; Philips Healthcare, Best, The Netherlands) equipped with a 28-channel coil for cardiac imaging. The protocol comprised REACT (index test), CE-MRA (reference standard), as well as 2D bSSFP breath-hold cine sequences in standard orientations (4-chamber, 2-chamber, 3-chamber, short axis, and aortic sinus), and a 2D bSSFP MRA of the abdominal aorta.

#### REACT

The specifics of the REACT sequence have been described in previous publications (29, 32, 33). In brief, a “modified” REACT approach was employed, which involves the use of a 30 ms T2 preparation pulse with a two-point DIXON (mDIXON XD; Philips Healthcare) readout (32). Given that background suppression of mDIXON XD with T2 preparation was deemed adequate for cardiovascular imaging (29, 32), no inversion-recovery prepulse was applied, in contrast to the original REACT sequence as described by Yoneyama et al. (25). Therefore, the modified REACT approach can be regarded as equivalent to T2 prepared Dixon non-CE-MRA (35). Furthermore, to compensate for cardiac and respiratory motion, ECG-triggering (end-diastolic) and respiratory navigator-triggering (diaphragmatic pencil-beam navigator, 6 mm gating window) were used. The acquisition of REACT was conducted in the coronal plane with immediate image reconstruction. Given the known fat-water swapping artifacts of the mDIXON XD readout, water-only, in-phase, out-of-phase, and fat-only images were reconstructed (29, 32, 33). To accelerate the acquisition of images, Compressed SENSE (Philips Healthcare), which combines compressed sensing and parallel imaging using SENSitivity Encoding (SENSE, Philips Healthcare), with a factor of 9 was employed (36). Datasets were acquired with using a Cartesian pseudo random k-space sampling scheme with high sampling density in the center of the k-space and lower sampling density towards the k-space periphery, resulting in a balanced variable density incoherent sampling pattern, which is acquired with low-high profile order, where every shot starts close to the k-space center. Finally, an iterative reconstruction based on L1 norm minimization in combination with regularization by the coil sensitivity distribution and SENSE parallel imaging was employed. Data sparsity was enforced by wavelet transformation and data consistency was ensured at each iteration.

#### CE-MRA

For CE-MRA, a radiofrequency-spoiled T1-weighted gradient echo sequence was employed. Gadobutrol (Gadovist; Bayer HealthCare Pharmaceuticals, Berlin, Germany; 0.1 ml/kg body weight) was administered at a weight based volume at a flow rate of 2,0 ml/s, immediately followed by a 20 ml saline flush at a flow rate of 2.0 ml/s into an antecubital vein. After determining the optimal time point for acquisition using a bolus-tracking sequence, the operator manually started the sequence and patients were instructed to perform an end-expiratory breath-hold. No ECG- or respiratory synchronization was used. Images were created by subtraction of a native and a CE acquisition. Acceleration of CE-MRA was performed using SENSE employing a factor of 4. Detailed imaging parameters of MRA sequences are given in Table 1.

**Table 1:** Scan parameters of CE-MRA, contrast-enhanced magnetic resonance angiography and REACT, relaxation-enhanced angiography without contrast and triggering.

Scan parameters	CE-MRA	REACT
K-space trajectory	Cartesian	Cartesian
Acquisition orientation	Coronal	Coronal
Acquired voxel size	1.20 × 1.39 × 3.60 mm^3^	1.69 × 1.70 × 1.70 mm^3^
Acquisition matrix size	376 × 286 × 44	236 × 299 × 100
Reconstructed voxel size	0.67 × 0.67 × 1.80 mm^3^	0.79 × 0.79 × 0.85 mm^3^
Field of view (FH × RL × AP)	450 × 396 × 157 mm^3^	400 × 508 × 170 mm^3^
T2 preparation	n/a	50 ms, refocusing pulses: 4
Repetition time	3.4 ms	6.0 ms
Echo time (1/2)	1.12 ms	1.69/3.8 ms
Flip angle	35°	15°
k-space lines per heartbeat	n/a	35
Acceleration factor	SENSE 4	Compressed SENSE 9
Image reconstruction	Real time	Immediate
Total acquisition time	02:58 ± 00:51 min	05:42 ± 00:38 min

FH, feet head; RL, right left; AP, anterior posterior.

### Image analysis

Two radiologists, one resident with three years of experience (reader 2), and one board-certified radiologist with seven years of experience in cardiovascular MRI (reader 1), evaluated anonymized datasets of REACT and CE-MRA in random order during separate reading sessions. Both readers reviewed the images independently, blinded to each other's results. Furthermore, a four-week interval between both MRA sequences was maintained to minimize the potential for recall bias. Readers were aware of potential fat-water swapping artifacts of REACT and free to choose between water-only, in-phase, out-of-phase, and fat-only images.

#### Aortic diameter measurements

Aortic diameter measurements (inner edge to inner edge approach) were performed at the following five levels: mid-graft, distal anastomosis, ascending aorta (at the level of the pulmonary trunk), aortic arch (between the branching of the left common carotid and the left subclavian artery); and at the descending aorta (at the level of the left atrium) (3, 11). All measurements were conducted on source images using the Multiplanar-Reconstruction-(MPR) tool in a commercially available image viewer (*DeepUnity Diagnost*, release 1.1.1.1, Dedalus Healthcare Systems Group, Bonn, Germany). At each level, measurements were performed in two orthogonal axes (maximum and minimum diameter) perpendicular to the vessel axis. For intraobserver agreement 25 randomly selected cases were reanalyzed six months after the initial assessment by reader 1.

#### Aortic dissection

Readers evaluated MRA datasets for the presence of AD and scored each dissection based on their location (Stanford type A, Stanford type B) (5). Furthermore, diagnostic confidence for evaluation of AD was rated on a 5-point Likert scale (1: non-diagnostic, 2: low confidence, 3: moderate confidence, 4: good confidence, 5: excellent confidence). Additionally, the two readers assessed the delineation of AD on a 5-point Likert scale (1: non-diagnostic, 2: poor delineation, 3: moderate delineation, 4: good delineation, 5: excellent delineation).

#### Image quality

Vessel quality of MRA datasets was rated at the above referenced five aortic levels using a 5-point Likert scale (1: non-diagnostic, image quality insufficient for diagnosis; 2: poor, inferior image quality; 3: fair, mediocre image quality; 4: good, image quality applicable for confident diagnosis; and 5: excellent, image quality yielding highly confident diagnosis).

#### Artifacts

Susceptibility artifacts (defined as a signal loss in all images of REACT or in CE-MRA images adjacent to surgical or interventional material) were rated at the above referenced five aortic levels on a 5-point Likert scale (1: non-diagnostic, 2: pronounced effect on image quality, 3: moderate effect on image quality, 4: slight effect on image quality, and 5: no impairment of image quality).

Motion artifacts (defined by blurring and reduced delineation of the vessel wall in MRA datasets) were evaluated at the above referenced five aortic levels on a 5-point Likert scale (1: non-diagnostic, 2: pronounced effect on image quality, 3: moderate effect on image quality, 4: slight effect on image quality, and 5: no impairment of image quality).

#### Fat-water swapping artifacts

Given the fact that fat-water-swapping artifacts only occur in REACT (37, 38), both readers assessed respective datasets for the presence of these artifacts (defined as a vascular signal loss in the water map with corresponding hyperintense signal in the fat map) in consensus.

### Statistics

Statistical analysis was performed by using GraphPad Prism version 10.2.3 for Mac OS X (GraphPad Software) and R programming language v. 4.0.2 with the open-source software RStudio (https://www.posit.co). Categorical variables are presented as frequencies and corresponding percentages. Subjective ratings are presented as frequencies and corresponding percentages and median with interquartile range. Continuous variables are indicated as the mean ± standard deviation and minimum to maximum. Normal distributions (ND) were checked with Shapiro-Wilk test. Differences were compared with Wilcoxon signed-rank tests (if not ND) or paired *t*-tests (if ND) and calculated per reader as well as by combining the measurements of all readers. Kendall's τ was calculated to assess interobserver agreement for subjective ratings (≤0.3 negligible, 0.31–0.5 low, 0.51–0.7 moderate, 0.71–0.9 high, and 0.91–1.00 very high) and ICC (two-way mixed-effects model for single measurements) was used for intra- and interobserver agreement as well as intersequence comparison of aortic diameters (<0.5 low, 0.5–0.75 moderate, 0.75–0.9 good, >0.9 excellent). Bland-Altman analysis was performed to evaluate the agreement between aortic diameters obtained from CE-MRA and REACT (pooled for both readers and separately for each reader), as well as the intra- and interobserver agreement between reader 1 and reader 2 for each imaging sequence (39). Maximum and minimum aortic diameters were analyzed separately. Differences between diameters are displayed as mean ± standard deviation and 95% limits of agreement. For all tests, a two-tailed *p*-value of <.05 was considered statistically significant.

## Results

### Study population

The final study population consisted of 58 cases in 34 patients [mean age of 45.64 ± 11.13 years, a mean BMI of 25.5 ± 4.7 kg/m^2^, and 31 female cases (53.4%)]. Aortic surgery was performed due to Stanford type A AD in 34 cases (58.6%) and thoracic aorta aneurysm in 24 cases (41.4%). Connective tissue disease was present in 47 cases [81.0%; MFS 32 cases (55.2%), Ehlers-Danlos syndrome in 15 cases (25.9%)].

David procedure was performed in 22 (37.9%) cases, Bentall procedure in 23 (39.7%) cases, supracoronary ascending aorta replacement in 9 cases (16.1%) cases, and supracoronary ascending aorta replacement with frozen elephant trunk in 4 (6.9%) cases. Additional aortic stent grafts of the descending aorta were implanted in 11 (18.9%) cases.

Detailed information about the study population is presented in Table 2.

**Table 2:** Study population and patient characteristics.

Patient characteristics	*n* (number)	% (percentage)
Age (years, mean ± SD)	45.64 ± 11.1	
BMI (mean ± SD)	25.5 ± 4.7	
Gender		
Female	31	53.4
Male	27	46.5
Patient characteristics		
Hypertension	29	50.0
Diabetes mellitus	5	8.6
Dyslipidemia	10	17.2
Smoking	9	15.5
Cardiac arrhythmia	24	41.3
Previous cardiac surgery	3	5.1
Indication for surgery		
Stanford type A aortic dissection	34	58.6
Thoracic aorta aneurysm	24	41.4
Connective tissue disease		
Marfan syndrome	32	55.2
Ehlers-Danlos syndrome	15	25.9
none	11	18.9
Surgical procedure		
Bentall procedure	23	39.7
David procedure	22	37.9
Supracoronary ascending aortic replacement	9	15.5
Supracoronary ascending aortic replacement with frozen elephant trunk	4	6.9
Stent graft of the descending aorta	11	18.9

SD, standard deviation; *n*, numbers; BMI, body mass index.

### Magnetic resonance imaging

REACT yielded an average total acquisition time of 05:42 ± 00:38 min, which was depending on the patient's breathing frequency and heart rate. CE-MRA had a total acquisition time of 02:58 ± 00:51 min (*p* < .001), when considering the time needed for bolus-tracking sequence, reconstruction, and subtraction of the pre-contrast mask. All studies were performed without periprocedural complications.

### Image analysis

The two readers evaluated 116 datasets, comprising 58 REACT and 58 CE-MRA images, respectively.

#### Aortic diameter measurements

**Figure 2:**
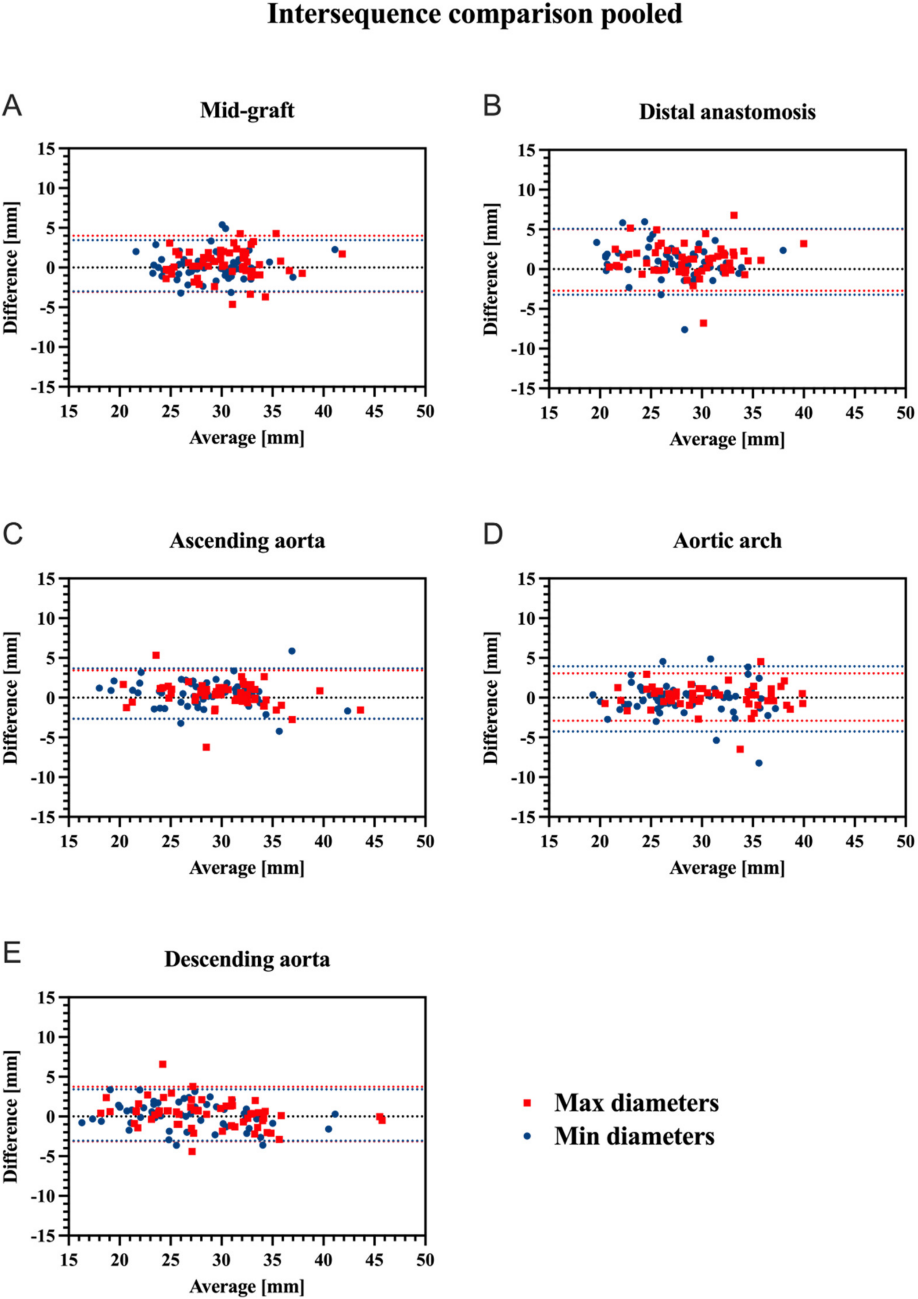
Bland-Altman plots for comparison of CE-MRA and REACT regarding maximum (red) and minimum (blue) aortic diameters at the different aortic levels pooled for both readers (mid-graft **(A)**, distal anastomosis **(B)**, ascending aorta **(C)**, aortic arch **(D)**, and descending aorta **(E)** upper and lower red/blue dotted lines indicate the corresponding 95% limits of agreement. CE-MRA, contrast-enhanced magnetic resonance angiography; REACT, relaxation-enhanced angiography without contrast and triggering.

Overall, vessel diameters were slightly larger in CE-MRA compared to REACT, albeit without statistical significance for most of the levels. Only at the distal anastomosis (maximum diameter: CE-MRA: 29.36 ± 4.15 mm vs. REACT: 28.23 ± 4.14 mm, *p* < .001; minimum diameter: 27.58 ± 3.85 vs. 26.66 ± 4.21 mm, *p* = .002) and the ascending aorta (minimum diameter: 28.67 ± 4.59 mm vs. 28.15 ± 4.77 mm, *p* = .02), CE-MRA yielded significantly larger diameters. Overall, the agreement between CE-MRA and REACT was good (e.g., maximum diameter of the distal anastomosis measured by reader 1: ICC=0.82) to excellent (e.g., maximum diameter of the descending aorta measured by reader 2: ICC = 0.97). Maximum and minimum diameters at the different levels pooled for both readers are presented in Table 3. Bland–Altman comparison between sequences pooled for both readers is given in Figure 2. Regarding the interobserver agreement for all levels of measurement, there was an excellent agreement (ICC > 0.9) for both methods. However, CE-MRA yielded a slightly lower interobserver agreement than REACT. Tables 4, 5 give detailed information about the interobserver agreement of aortic diameters in both MRA sequences. Furthermore, the intraobserver agreement was good for the maximum diameter at the mid-graft (CE-MRA: ICC = 0.86; REACT ICC = 0.85) and excellent for all other levels in both sequences (ICC > 0.9). Additional Bland-Altman analyses and detailed results of the inter- and intraobserver agreement and the intersequence agreement of the individual readers are provided in the supplementary data (Supplementary Figures S1–S6 and Supplementary Tables S1–S4).

**Table 3:** Maximum and minimum aortic diameters pooled for both readers of CE-MRA compared to REACT.

Intersequence comparison pooled			Mid-graft	Distal anastomosis	Ascending aorta	Aortic arch	Descending aorta
Maximum diameter	CE-MRA	Mean ± SD [mm]	30.63 ± 3.76	29.36 ± 4.15	30.25 ± 4.31	30.55 ± 5.10	28.65 ± 5.51
	REACT	Mean ± SD [mm]	30.17 ± 3.67	28.23 ± 4.14	29.85 ± 4.67	30.47 ± 5.17	28.36 ± 6.01
	Difference	Mean ± SD [mm]	0.46 ± 1.81	1.14 ± 1.97	0.39 ± 1.56	0.08 ± 1.5	0.29 ± 1.75
		95% Limits of agreement [mm]	−3.08–4.00	−2.73–5.01	−2.66–3.45	−2.91–3.07	−3.14–3.73
	ICC (95% CI)		0.8614 (0.7765–0.9156)	0.8187 (0.7117–0.8886)	0.8872 (0.8165–0.9316)	0.9417 (0.9035–0.9651)	0.9222 (0.8719–0.9532)
	*p* (*t*-test)		.06	**<.001**	.06	.69	.21
Minimum diameter	CE-MRA	Mean ± SD [mm]	28.95 ± 3.67	27.58 ± 3.85	28.67 ± 4.59	28.23 ± 4.65	27.02 ± 5.28
	REACT	Mean ± SD [mm]	28.73 ± 3.60	26.66 ± 4.21	28.15 ± 4.77	28.38 ± 4.78	26.85 ± 5.68
	Difference	Mean ± SD [mm]	0.22 ± 1.64	0.92 ± 2.12	0.52 ± 1.62	−0.15 ± 2.09	0.17 ± 1.65
		95% Limits of agreement [mm]	−3.0–3.44	−3.23–5.07	−2.65–3.69	−4.25–3.95	−3.07–3.40
	ICC (95% CI)		0.8978 (0.8331–0.9382)	0.8624 (0.7781–0.9162)	0.9404 (0.9013–0.9643)	0.9027 (0.8410–0.9413)	0.9549 (0.9250–0.9731)
	*p* (*t*-test)		.32	**.002**	**.02**	.59	.45

Bold indicates statistical significance; CE-MRA, contrast-enhanced magnetic resonance angiography; CI, confidence interval; ICC, intraclass correlation coefficient; REACT, relaxation-enhanced angiography without contrast and triggering; SD, standard deviation.

**Table 4:** Interobserver agreement between both readers for maximum and minimum diameters of measured aortic levels using REACT.

Interobserver agreement REACT			Mid-graft	Distal anastomosis	Ascending aorta	Aortic arch	Descending aorta
Maximum diameter	Reader 1	Mean ± SD [mm]	30.06 ± 3.66	28.15 ± 4.17	29.85 ± 4.77	30.33 ± 5.16	28.20 ± 6.08
	Reader 2	Mean ± SD [mm]	30.31 ± 3.70	28.30 ± 4.12	29.87 ± 4.66	30.63 ± 5.19	28.59 ± 5.99
	Difference	Mean ± SD [mm]	−0.25 ± 0.47	−0.15 ± 0.32	−0.02 ± 1.33	−0.30 ± 0.79	−0.39 ± 0.67
		95% Limits of agreement [mm]	−1.18–0.68	−0.77–0.47	−2.62–2.58	−1.84–1.24	−1.70–0.92
	ICC (95% CI)		0.9917 (0.9860–0.9951)	0.9971 (0.9950–0.9983)	0.9610 (0.9350–0.9767)	0.9885 (0.9806–0.9932)	0.9938 (0.9896–0.9964)
	*p* (*t*-test)		**<.001**	**<.001**	.91	**.006**	**<.001**
Minimum diameter	Reader 1	Mean ± SD [mm]	28.60 ± 3.54	26.64 ± 4.20	28.01 ± 4.69	28.31 ± 4.80	26.74 ± 5.68
	Reader 2	Mean ± SD [mm]	28.82 ± 3.67	26.67 ± 4.22	28.28 ± 4.95	28.43 ± 4.78	26.90 ± 5.65
	Difference	Mean ± SD [mm]	−0.22 ± 0.61	−0.03 ± 0.23	−0.28 ± 1.40	−0.12 ± 0.46	−0.16 ± 0.59
		95% Limits of agreement [mm]	−1.42–0.98	−0.48–0.42	−3.02–2.47	−1.02–0.77	−1.31–1.00
	ICC (95% CI)		0.9855 (0.9757–0.9914)	0.9985 (0.9975–0.9991)	0.9579 (0.9299–0.9749)	0.9954 (0.9923–0.9973)	0.9946 (0.9909–0.9968)
	*p* (*t*-test)		**.008**	.36	.14	**.04**	**.049**

Bold indicates statistical significance; CI, confidence interval; ICC, intraclass correlation coefficient; REACT, relaxation-enhanced angiography without contrast and triggering; SD, standard deviation.

**Table 5:** Interobserver agreement between both readers for maximum and minimum diameters of measured aortic levels using CE-MRA.

Interobserver agreement CE-MRA			Mid-graft	Distal anastomosis	Ascending aorta	Aortic arch	Descending aorta
Maximum diameter	Reader 1	Mean ± SD [mm]	30.94 ± 4.04	29.77 ± 4.17	30.36 ± 4.47	30.78 ± 4.97	28.98 ± 5.53
	Reader 2	Mean ± SD [mm]	30.41 ± 3.66	29.00 ± 4.28	30.18 ± 4.31	30.35 ± 5.24	28.34 ± 5.60
	Difference	Mean ± SD [mm]	0.54 ± 1.55	0.78 ± 1.59	0.18 ± 1.48	0.43 ± 0.91	0.63 ± 1.50
		95% Limits of agreement [mm]	−2.49–3.57	−2.34–3.89	−2.73–3.08	−1.35–2.21	−2.31–3.57
	ICC (95% CI)		0.9194 (0.8676–0.9515)	0.9293 (0.8834–0.9575)	0.9431 (0.9057–0.9659)	0.9842 (0.9735–0.9906)	0.9637 (0.9394–0.9783)
	*p* (*t*-test)		**.01**	**<.001**	.37	**<.001**	**.002**
Minimum diameter	Reader 1	Mean ± SD [mm]	28.99 ± 3.88	27.73 ± 3.82	28.59 ± 4.73	28.28 ± 4.64	27.18 ± 5.20
	Reader 2	Mean ± SD [mm]	28.82 ± 3.55	27.38 ± 3.95	28.70 ± 4.54	28.14 ± 4.84	26.84 ± 5.41
	Difference	Mean ± SD [mm]	0.17 ± 1.29	0.35 ± 0.81	−0.10 ± 1.61	0.14 ± 1.72	0.34 ± 1.16
		95% Limits of agreement [mm]	−2.35–2.69	−1.24–1.94	−3.25–3.04	−3.23–3.51	−1.94–2.61
	ICC (95% CI)		0.9402 (0.9010–0.9642)	0.9782 (0.9634–0.9870)	0.9408 (0.9021–0.9646)	0.9349 (0.8924–0.9609)	0.9761 (0.9599–0.9858)
	*p* (*t*-test)		.31	**.002**	.63	.54	**.03**

Bold indicates statistical significance; CI, confidence interval; CE-MRA, contrast-enhanced magnetic resonance angiography; ICC, intraclass correlation coefficient; SD, standard deviation.

#### Aortic dissection

Both readers detected a total of 25 residual AD in 58 cases (43.1%) in both MRA sequences. Stanford type A was identified in 21 cases (84.0%) with Stanford type B being observed in four cases (16.0%). There was neither a significant difference regarding the diagnostic confidence [CE-MRA 4.0 (3.0–4.0) vs. REACT 4.0 (3.0–4.0), *p* = .81] nor regarding the delineation of AD between CE-MRA and REACT [4.0 (3.0–4.5) vs. 4.0 (3.0–4.0), *p* = .41].

#### Image quality

**Figure 3:**
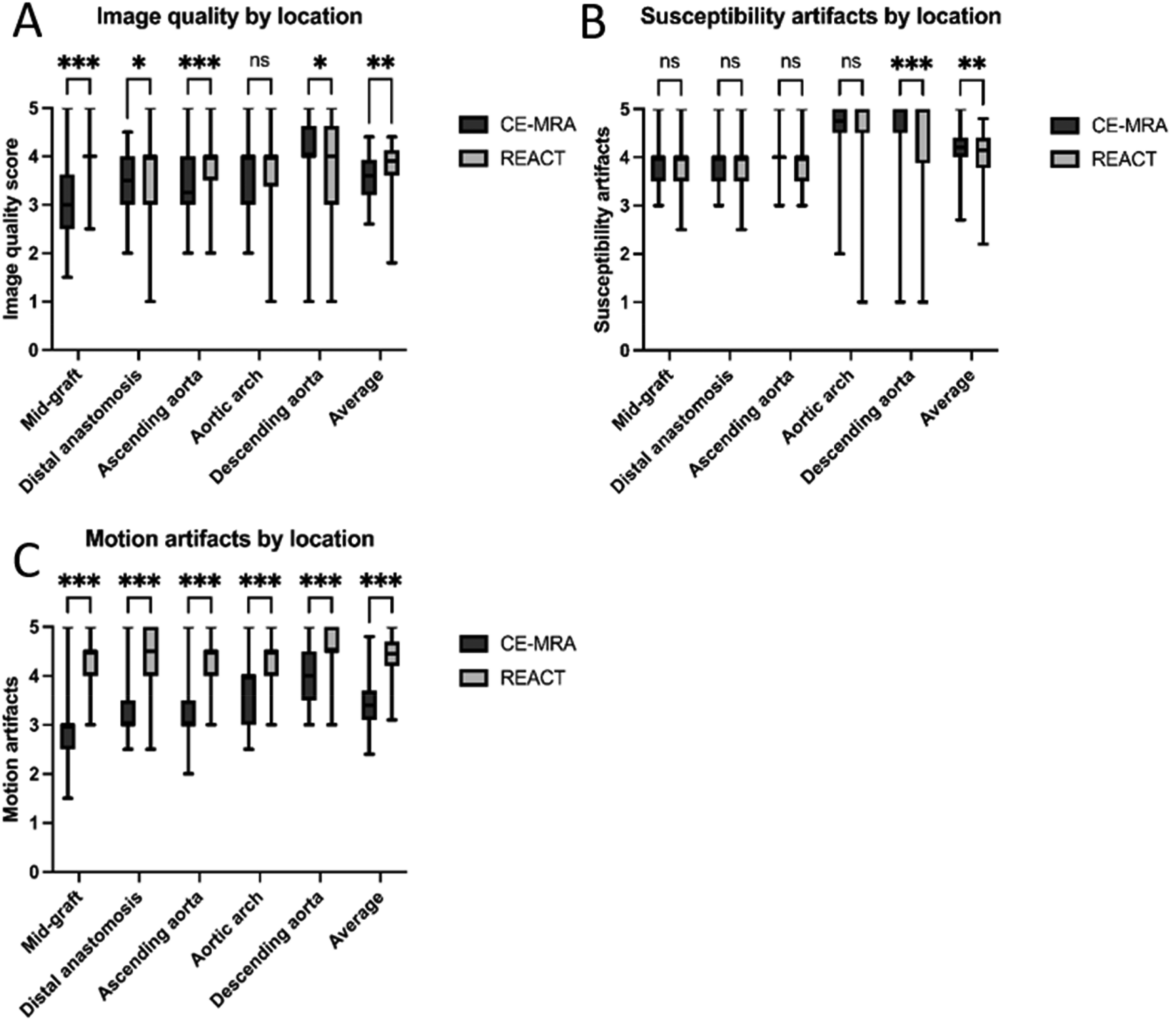
Box plots for subjective image quality **(A)**, susceptibility artifacts **(B)**, and motion artifacts **(C)** for CE-MRA compared to REACT at each aortic level. All values displayed are based on the pooled ratings of both readers. ns = *p* > .05; *=*p* < .05; **=*p* < .01; ***=*p* < .001. CE-MRA, contrast-enhanced magnetic resonance angiography; REACT, relaxation-enhanced angiography without contrast and triggering.

Overall, image quality for all aortic levels was superior in REACT than in CE-MRA [CE-MRA: 3.6 [3.2–3.9] vs. REACT 3.9 [3.6–4.1], *p* = .02] with mid-graft [3.0 (2.5–3.6) vs. 4.0 (4.0–4.0), *p* < .001], distal anastomosis [3.5 (3.0–4.0) vs. 4.0 (3.0–4.0), *p* = .02], and ascending aorta [3.25 (3.0–4.0) vs. 4.0 (3.5–4.0), *p* < .001], yielding significant higher scores. At the level of the descending aorta, CE-MRA showed significant higher image quality scores than REACT [4.0 (4.0–4.6) vs. 4.0 (3.0–4.6), *p* = .04]. Of note, one patient (three cases) with extensive susceptibility artifacts after spinal fusion yielded an image quality score of 1 in the REACT in all cases, while the CE-MRA achieved scores of 3, 3, and 4. Excluding this outlier, no difference in image quality of the descending aorta was observed [4.0 (4.0–5.0) vs. 4.0 (3.5–5.0); *p* = .15]. Detailed image quality results are given in Table 6, the comparison of image quality separated by location is presented in Figure 3.

**Table 6:** Subjective image quality in CE-MRA and REACT pooled for both readers.

Image quality	CE-MRA	REACT	*p* (wilcoxon)
	Median [IQR]	Median [IQR]	
Average	3.6 [3.2–3.93]	3.9 [3.6–4.13]	**.** **002**
Mid-graft	3 [2.5–3.63]	4 [4–4]	**<** **.** **001**
Distal anastomosis	3.5 [3–4]	4 [3–4]	**.** **02**
Ascending aorta	3.25 [3–4]	4 [3.5–4]	**<** **.** **001**
Aortic arch	4 [3–4]	4 [3.38–4]	.12
Descending aorta	4 [4–4.63]	4 [3–4.63]	**.** **04**
Kendall's τ	0.69	0.69	

Kendall's τ indicates the agreement between the two readers across all levels. Bold indicates statistical significance. CI, confidence interval; IQR, interquartile range; CE-MRA, contrast-enhanced magnetic resonance angiography; REACT, relaxation-enhanced angiography without contrast and triggering.

#### Artifact scoring

Overall, susceptibility artifacts yielded a slightly higher impact in REACT than in CE-MRA [4.2 (4.0–4.4) vs. 4.1 (3.8–4.4), *p* = .003]. While there were no differences at the proximal levels of the thoracic aorta adjacent to surgical material, the descending aorta was significantly affected by susceptibility artifacts in REACT compared to CE-MRA [5.0 (4.5–5.0) vs. 5.0 (3.8–4.4), *p* < .001]. Detailed information about susceptibility artifacts is presented in Table 7 and Figure 3. Motion artifacts were more severe in CE-MRA than in REACT pooled for all levels [3.4 (3.1–3.7) vs. 4.5 (4.2–4.7), *p* < .001] as well as for each level separately (all *p* < .001). In particular, mid-graft, distal anastomosis, and ascending aorta showed substantial differences between both techniques. Detailed information about motion artifacts are presented in Table 8 and Figure 3.

**Table 7:** Susceptibility artifacts in CE-MRA and REACT pooled for both readers.

Susceptibility artifacts	CE-MRA	REACT	*p* (wilcoxon)
	Median [IQR]	Median [IQR]	
Average	4.2 [4–4.4]	4.15 [3.78–4.4]	**.** **003**
Mid-graft	4 [3.5–4]	4 [3.5–4]	.27
Distal anastomosis	4 [4–4]	4 [3.5–4]	.09
Ascending aorta	4.5 [4.75–5]	4 [3.5–4]	.44
Aortic arch	5 [4.5–5]	5 [4.5–5]	.16
Descending aorta	4.2 [4–4.4]	5 [3.88–5]	**<** **.** **001**
Kendall's τ	0.55	0.58	

Kendall's τ indicates the agreement between the two readers across all levels. Bold indicates statistical significance. CI, confidence interval; IQR, interquartile range; CE-MRA, contrast-enhanced magnetic resonance angiography; REACT, relaxation-enhanced angiography without contrast and triggering.

**Table 8:** Motion artifacts in CE-MRA and REACT pooled for both readers.

Motion artifacts	CE-MRA	REACT	*p* (wilcoxon)
	Median [IQR]	Median [IQR]	
Average	3.4 [3.1–3.7]	4.45 [4.2–4.7]	**<** **.** **001**
Mid-graft	3 [2.5–3]	4.5 [4–4.5]	**<** **.** **001**
Distal anastomosis	3 [3–3.5]	4.5 [4–5]	**<** **.** **001**
Ascending aorta	3 [3–3.5]	4.5 [4–4.5]	**<** **.** **001**
Aortic arch	4 [3–4]	4.5 [4–4.5]	**<** **.** **001**
Descending aorta	4 [3.5–4.5]	4.5 [4.5–5]	**<** **.** **001**
Kendall's τ	0.50	0.29	

Kendall's τ indicates the agreement between the two readers across all levels. Bold indicates statistical significance. CI, confidence interval; IQR, interquartile range; CE-MRA, contrast-enhanced magnetic resonance angiography; REACT, relaxation-enhanced angiography without contrast and triggering.

#### Fat-water swapping artifacts

**Figure 4:**
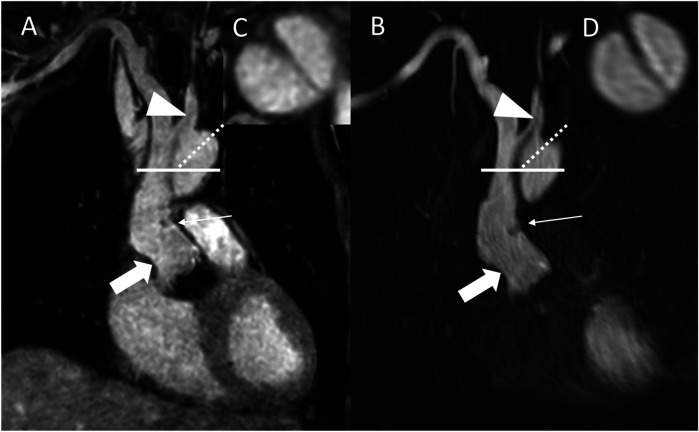
REACT [**(A)**, source images, water-only, coronal plane] and CE-MRA [**(B)**, source images, coronal plane] in a 54-year-old male after bentall procedure due to Stanford type A aortic dissection (AD). The delineation of the aortic graft (wide arrow: mid graft, thin arrow: distal anastomosis) and remaining AD affecting the aortic arch and the left common carotid artery (arrowhead) is comparable in both sequences. The axial reformation [square; REACT: **(C)** CE-MRA: **(D)**] serves to highlight the delineation of the dissection membrane in both sequences. CE-MRA, contrast-enhanced magnetic resonance angiography; REACT, relaxation-enhanced angiography without contrast and triggering.

**Figure 5:**
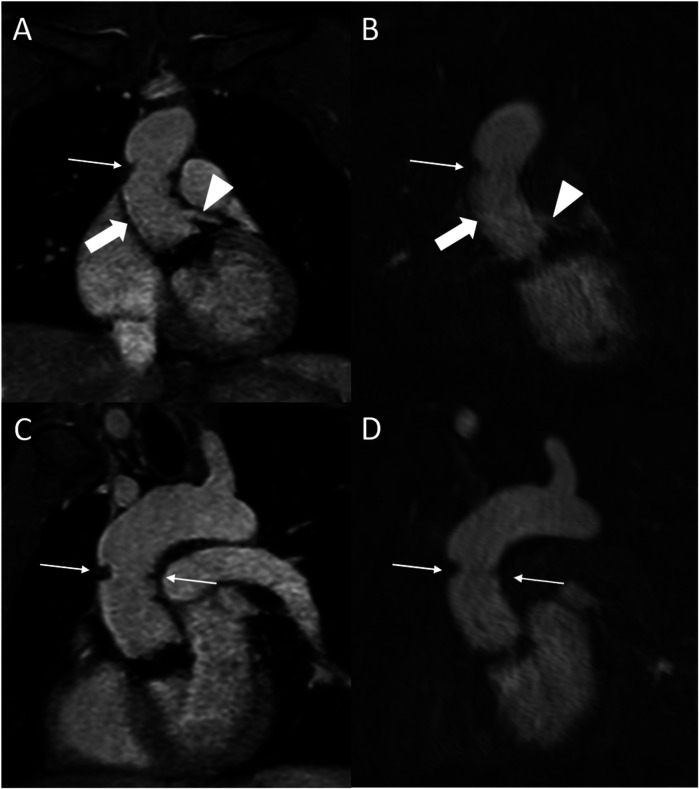
REACT [source images, water-only, coronal **(A)** and parasagittal planes **(C)**] and CE-MRA [source images, coronal **(B)** and parasagittal planes **(D)**] in a 27-year-old male after Bentall procedure due to Stanford type A aortic dissection. Note the superior delineation of the aortic graft (wide arrow: mid graft) and the left coronary artery (arrowhead) in REACT due to motion artifacts in CE-MRA. While the distal anastomosis and suture lines can be delineated in both sequences (thin arrows), REACT yields a superior delineation of these structures due to above mentioned artifacts in CE-MRA. CE-MRA, contrast-enhanced magnetic resonance angiography; REACT, relaxation-enhanced angiography without contrast and triggering.

**Figure 6:**
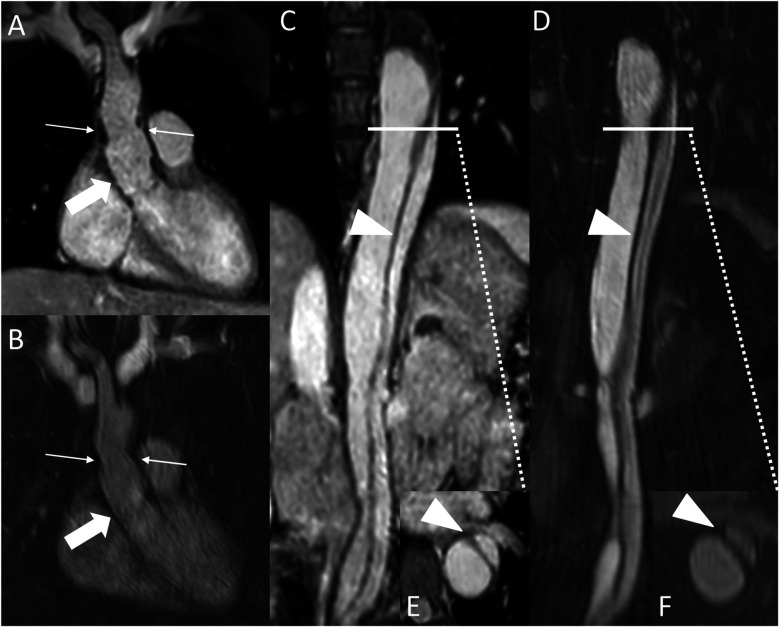
REACT [**(A)**, source image, water-only, coronal plane] and CE-MRA [**(B)**, coronal plane] in a 44-year-old female after david-procedure due to Stanford type A aortic dissection. Note the higher vessel contrast at the level of the aortic graft (wide arrow: mid graft, thin arrow: distal anastomosis) in REACT due to mistiming in CE-MRA. The residual AD (arrowheads) affecting the descending and abdominal aorta can be equally delineated in both MRA sequences [REACT: **(C)** paracoronal reformation, **(E)** axial reformation; CE-MRA: **(D)** coronal reformation, **(F)** axial reformation]. CE-MRA, contrast-enhanced magnetic resonance angiography; REACT, relaxation-enhanced angiography without contrast and triggering.

**Figure 7:**
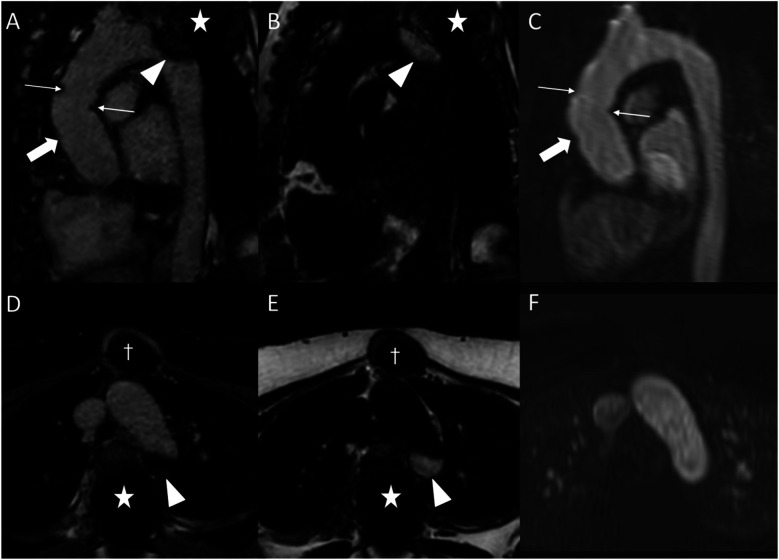
Fat-water separation artifact in REACT as shown in parasagittal reformations **(A–C)** and axial source images **(D–F)** in a 41-year-old female with Marfan syndrome after bentall procedure due to Stanford type A aortic dissection. While the REACT sequence [**(A)** and **(D)**, water-only images; **(B,E)** fat-only images] enables a superior delineation of the distal anastomosis (wide arrow: mid graft, thin arrows: distal anastomosis) compared to CE-MRA **(C,F)**, the aortic graft is apparent in both sequences. Furthermore, there is a signal loss at the aortic arch in the water-only images of REACT (arrowheads) due to spinal fusion (asterisk) with corresponding fat-only images showing a hyperintense signal, clarifying the drop-out as an artifact. Note that the susceptibility artifacts from sternal wires (daggers) do not hamper the image quality of REACT. CE-MRA, contrast-enhanced magnetic resonance angiography; REACT, relaxation-enhanced angiography without contrast and triggering.

**Figure 8:**
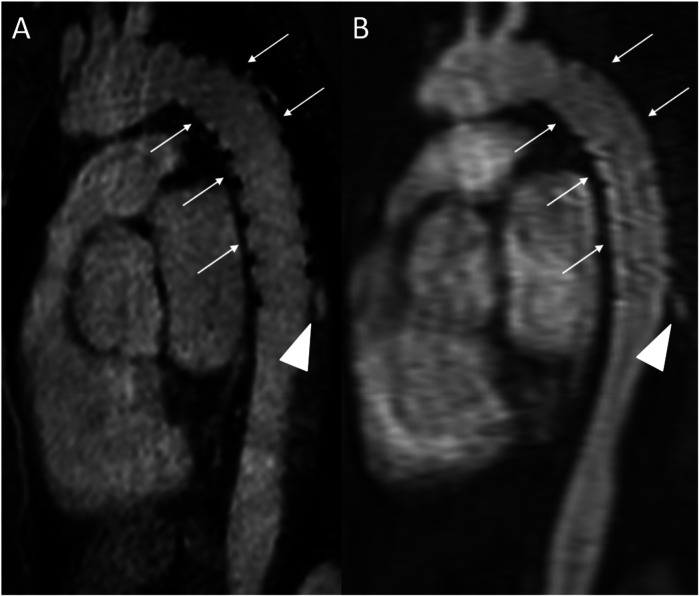
REACT [**(A)**, source image, water-only, parasagittal plane] and CE-MRA [**(B)**, parasagittal plane] in a 46-year-old female patient following supracoronary replacement of the ascending aorta and additional stent graft of the descending aorta due to Stanford type A aortic dissection involving the aortic arch and descending aorta. Note the stronger delineation of stent strats in REACT compared to CE-MRA (thin arrows). Both REACT and CE-MRA present residual inflow (arrowheads) of the false lumen. CE-MRA, contrast-enhanced magnetic resonance angiography; REACT, relaxation-enhanced angiography without contrast and triggering.

Overall, 27 fat-water swapping artifacts were observed, which occurred in 23 of the 58 cases (39.66%). These artifacts were found in the left subclavian artery (*n* = 14; 51.9%), the inferior vena cava (*n* = 9; 33.3%), the left brachiocephalic vein (*n* = 1; 3.7%), the left common carotid artery (*n* = 1; 3.7%), the descending thoracic aorta (*n* = 1; 3.7%), and the main pulmonary artery (*n* = 1; 3.7%). Of note, the majority of artifacts (*n* = 20; 74.1%) were not adjacent to surgical material. Corresponding to the focal signal loss in the water-only images, fat-only images provided a focal hyperintense signal within the vessel lumen in every case, clarifying the dropout as an artifact. Figures 4–8 give exemplary comparisons of REACT-non-CE-MRA and CE-MRA.

## Discussion

In this retrospective, single-center study, we performed an intraindividual comparison of a novel 3D isotropic flow independent non-CE-MRA technique (REACT) with CE-MRA in patients after aortic root replacement and/or ascending aortic surgery.

The major findings of this study are the following: 1. After aortic root replacement and/or ascending aortic surgery, REACT yielded superior image quality at the aortic graft and less motion artifacts while yielding slightly smaller diameters for the distal anastomosis and partly in the ascending aorta with higher interobserver agreement compared to CE-MRA. 2. While the impact of susceptibility artifacts did not differ between MRA at the aortic graft, they led to decreased image quality in REACT at the descending aorta. 3. In a short acquisition time, REACT enables an equivalent depiction of residual AD compared to CE-MRA.

The majority of prior studies investigating different non-CE-MRA techniques for imaging of the thoracic aorta, e.g., 2D bSSFP (24, 40), 3D bSSFP (41, 42), 2D quiescent interval slice-selective (QISS)-MRA (43), and Dixon-based approaches (30, 32, 33, 44) almost exclusively focused on preoperative imaging. In contrast, data after surgery is sparse and limited to bSSFP after ascending aortic surgery only 2D bSSFP (40) and QISS-MRA after abdominal endovascular repair (45, 46). Therefore, the knowledge about the performance of non-CE-MRA in these challenging patients with potentially decreased image quality due to surgical material is limited and needs further evaluation. In the present study, REACT showed higher image quality scores then CE-MRA at the aortic graft and ascending aorta. These findings are contrary to Veldhoen et al., who reported a similar image quality for bSSFP and CE-MRA in MFS patients after aortic root surgery (40). These results are mostly due to the susceptibility of bSSFP to off-resonance effects caused by B0 inhomogeneities and highly pulsatile flow, which typically manifest as banding artifacts and which are more pronounced in postoperative patients given the surgical material at the graft site and the surgical entryway (47–50). In contrast, the REACT sequence is widely insensitive to B0 inhomogeneities given its Dixon readout which provides robust suppression of fat and background and allows for separation of water and fat (25, 51), enabling sufficient depiction of the thoracic graft and the aortic root and/or ascending aorta. Additionally and as previously shown for 2D and 3D non-CE-MRA techniques, ECG-triggering and respiratory-gating of REACT enables the suppression of pulsation and breathing artifacts, which are more pronounced at the proximal aorta, with subsequently superior image quality compared to the untriggered CE-MRA (24, 32, 40, 52). Especially in patients after aortic surgery patients with potential pulmonary comorbidities, sternotomy and/or chest wall deformities, resulting in reduced lung capacity, the free-breathing approach of REACT proves to be beneficial since these patients are often unable to perform long breath-holds as required for high quality first-pass CE-MRA (53). These findings are in line with the studies by Isaak et al., who investigated REACT in congenital heart disease (CHD) in children and adults, primarily after surgery, and reported a superior image quality of REACT compared to first-pass CE-MRA and single-phase steady-state CE-MRA for the ascending aorta (30, 31).

These technical specificities regarding cardiac and respiratory synchronisation also explain the difference in aortic diameters with CE-MRA yielding significantly larger measurements than REACT at the distal anastomosis and partly in the ascending aorta with CE-MRA being hampered by pulsation and breathing artifacts and highly pulsatile flow leading to reduced vessel delineation and subsequently larger diameters. These findings are in line with results from other studies comparing non-CE-MRA techniques with untriggered CE-MRA, e.g., 2D bSSFP (24, 40), 3D bSSFP (42), and REACT (32, 33). Of note mid-graft, mostly due to its rigid structure, and descending aorta, likely given its decreased pulsatile flow compared to the ascending aorta, showed no difference between MRA techniques in the present study (24, 32). These findings are mostly in line with Veldhoen et al., who reported larger diameters in CE-MRA compared to 2D bSSFP after aortic surgery at all levels, especially at the distal anastomosis and the ascending aorta (40). In the present study, REACT yielded a higher interobserver agreement for aortic diameters compared to CE-MRA, which is consistent with Veldhoen et al. for postoperative bSSFP (40) and with Pennig et al. (32) for preoperative REACT, predominantly due to the superior delineation of the vessel wall in the non-CE-MRA technique given its cardiac and respiratory synchronisation.

Veldhoen et al. observed a higher amount of artifacts in bSSFP than in CE-MRA at the aortic graft albeit without reaching statistical significance, mainly due to the inherent limitations of bSSFP as outlined above (24, 40). In contrast, the REACT sequence, given its insensitivity to B0 inhomogeneities (47–50), was not affected by susceptibility artifacts at mid-graft, distal anastomosis, and ascending aorta in the present study, underlining its potential for unimpaired imaging of the aortic graft without gadolinium contrast. These results are in line with above referenced studies by Isaak et al. in CHD patients, who reported no difference in susceptibility artifacts between REACT and CE-MRA sequences (30, 31). However, in the present study, REACT was strongly impaired by susceptibility artifacts at the descending aorta in some cases. In particular, one patient (three cases) with extensive extravascular metallic material after spinal fusion, which presumably resulted in pronounced B0 inhomogeneities, was responsible for these outliers, indicating limitations of the REACT technique in such patients.

Fat-water swapping artifacts represent a common occurrence in Dixon-based imaging such as REACT (32, 33, 44). In the present study, these artifacts were observed in 40% of examinations, being slightly higher compared to patients after surgery for CHD (16%–33%) (30, 31) and mostly due to pronounced surgical material after aortic surgery as well as high or turbulent flow, resulting in an inappropriate allocation of signal in water-only and fat-only images. In this context, the inferior vena cava, as observed in above referenced studies, and the left subclavian artery, as shown in previous studies investigating REACT for cervical artery imaging (26–28), were predominantly affected. Even though affecting the thoracic aorta in only one case, it is pivotal to acquire all different images of REACT to circumvent these artifacts.

Despite its usage in patients after aortic surgery with possibly irregular breathing patterns, the REACT sequence enables the depiction of the whole thoracic aorta in 05:45 min, which is comparable to the application of REACT in CHD (7:00 min) and in MFS (5:00–6:30 min), predominantly prior to surgery (29, 32, 33). The combined time of acquisition and reconstruction of REACT is lower than for other 3D Dixon-based techniques accelerated by Compressed SENSE (8–10 min) (44) and 3D SSFP (up to 10 min) (11, 33, 34) while Veldhoen et al. did not report a precise image acquisition time for 2D bSSFP after aortic surgery (40). As reported for 2D bSSFP after aortic surgery (40), the REACT sequence in this study enabled the detection of all cases of residual aortic dissection with to CE-MRA comparable delineation and diagnostic confidence, highlighting the potential of REACT for postoperative assessment of the thoracic aorta with its 3D isotropic readout potentially facilitating vascular assessment as compared to 2D anisotropic non-CE-MRA approaches (24, 40, 54).

### Limitations

Besides its retrospective, single-center setting, several limitations have to be acknowledged in this study. Firstly, given the obvious differences in appearance of MRA techniques, readers were not blinded to the type of sequence potentially influencing the results. Secondly, patients with severe motion artefacts or technical failure in any MRA sequence were excluded, which could result in a selection bias.

Thirdly, the final study population was of moderate size and heterogeneous with different techniques for aortic root replacement and/or ascending aortic surgery, which hampers exact imaging recommendations for each procedure. Fourthly, no direct comparison to other non-CE-MRA techniques, e.g., QISS-MRA or bSSFP-MRA, was performed in this work, which could nurture future research. Fifthly, we regarded untriggered breath-hold 3D CE-MRA with inferior through-plane resolution as the reference standard, which may represent a limitation of this work given the fact that ECG-gating has shown to improve the image quality of CE-MRA. Nevertheless, previous studies evaluating ECG-gated CE-MRA showed that ECG-gating does not yield the same high image quality of ECG- and navigator gated 3D SSFP for the aortic root (19, 52). Sixthly, the chosen Compressed SENSE factor for REACT was based on studies performed in patients without surgical and/or interventional material (29, 32, 33) and based on our clinical experience but not after profound investigation of different undersampling factors. It is therefore possible that future research using artificial intelligence for image reconstruction may lead to a faster acquisition of REACT in patients after aortic surgery.

## Conclusions

REACT allows for robust and fast imaging of the thoracic aorta after aortic root replacement and/or ascending aortic surgery with superior image quality compared to CE-MRA at the proximal aortic levels without diagnostic compromise regarding the detection of AD. Although susceptibility and fat-water separation artifacts must be cautiously observed, this study indicates the feasibility of REACT for assessment of the thoracic aorta after ascending aortic surgery and expands its clinical use for gadolinium-free MRA to these patients.

## Data Availability

The original contributions presented in the study are included in the article/Supplementary Material, further inquiries can be directed to the corresponding author.
