# Supplementary material for: Assessment of the thoracic aorta after aortic root replacement and/or ascending aortic surgery using 3D relaxation-enhanced angiography without contrast and triggering
Source: Front Cardiovasc Med. 2025 Mar 12;12:1532661. doi: 10.3389/fcvm.2025.1532661 (PMC11937005; doi:10.3389/fcvm.2025.1532661)
Supplement: Supplementary file 1 [file Datasheet1.pdf]

Supplementary data

Frontiers in Cardiovascular Medicine

Assessment of the Thoracic Aorta after Aortic Root Replacement and/or Ascending Aortic Surgery Using 3D Relaxation-Enhanced Angiography without Contrast and Triggering

Authors: (blinded for submission)

Affiliations: (blinded for submission)

Corresponding author (*): (blinded for submission)

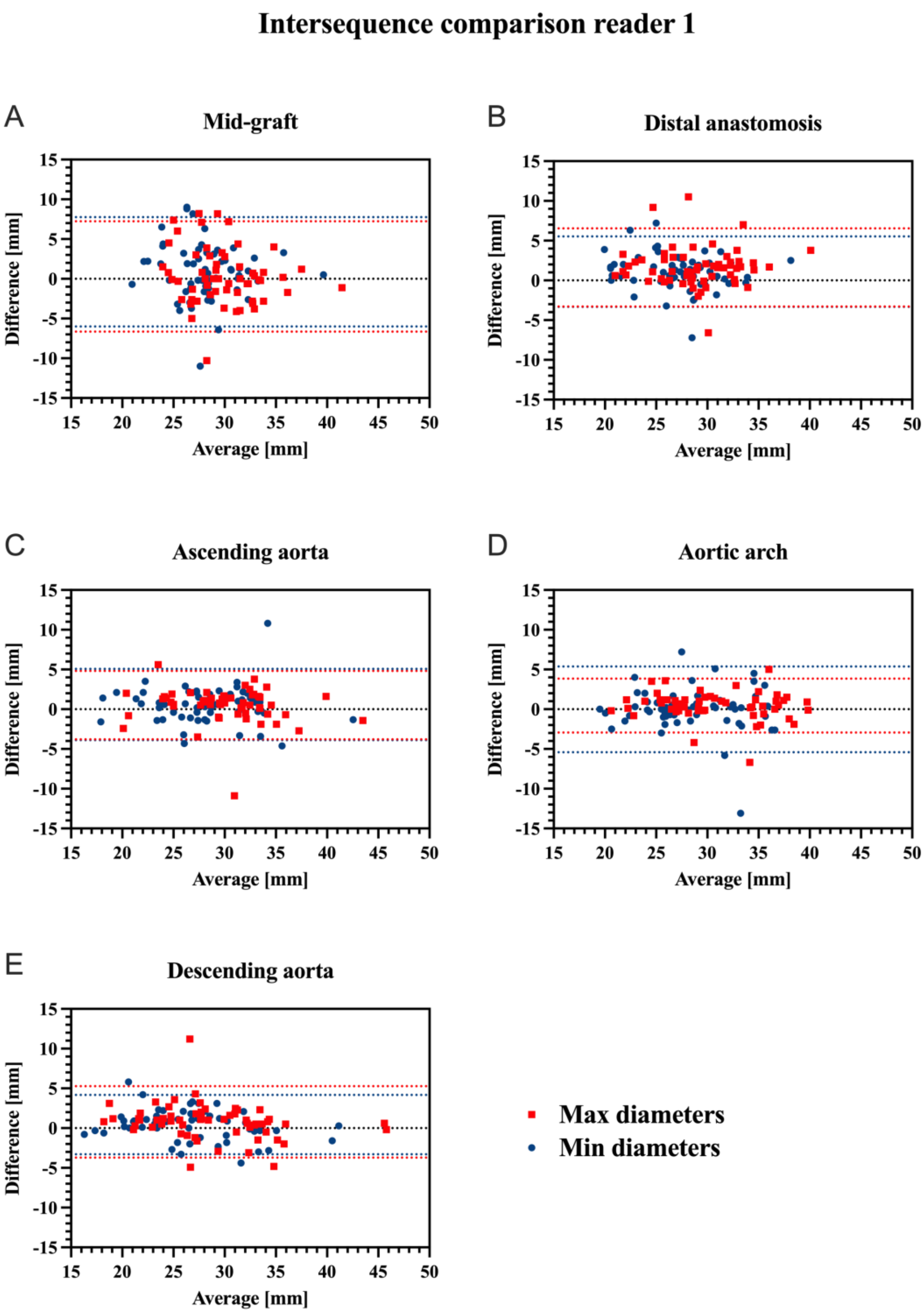

Suppl. figure 1: Bland-Altman plots for comparison of CE-MRA and REACT regarding maximum (red) and minimum (blue) aortic diameters at the different aortic levels measured by reader 1 (mid-graft (A), distal anastomosis (B), ascending aorta (C), aortic arch (D), and descending aorta (E). Upper and lower red/blue dotted lines indicate the corresponding 95% limits of agreement. CE-MRA=contrast-enhanced magnetic resonance angiography; REACT=Relaxation-Enhanced Angiography without Contrast and Triggering.

Intersequence comparison reader 2

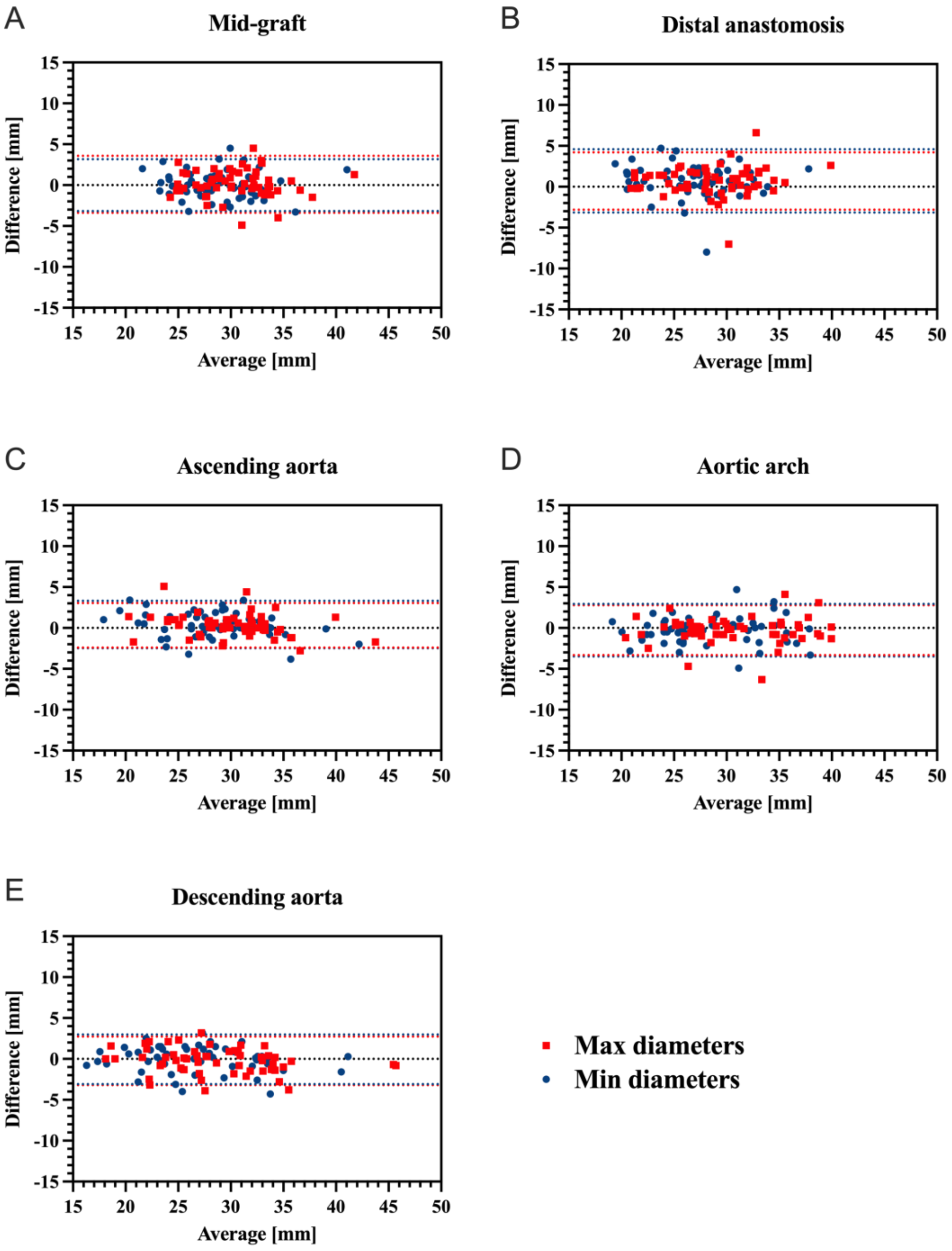

Suppl. figure 2: Bland-Altman plots for comparison of CE-MRA and REACT regarding maximum (red) and minimum (blue) aortic diameters at the different aortic levels measured by reader 2 (mid-graft (A), distal anastomosis (B), ascending aorta (C), aortic arch (D), and descending aorta (E). Upper and lower red/blue dotted lines indicate the corresponding 95% limits of agreement. CE-MRA=contrast-enhanced magnetic resonance angiography; REACT=Relaxation-Enhanced Angiography without Contrast and Triggering.

Interobserver agreement REACT

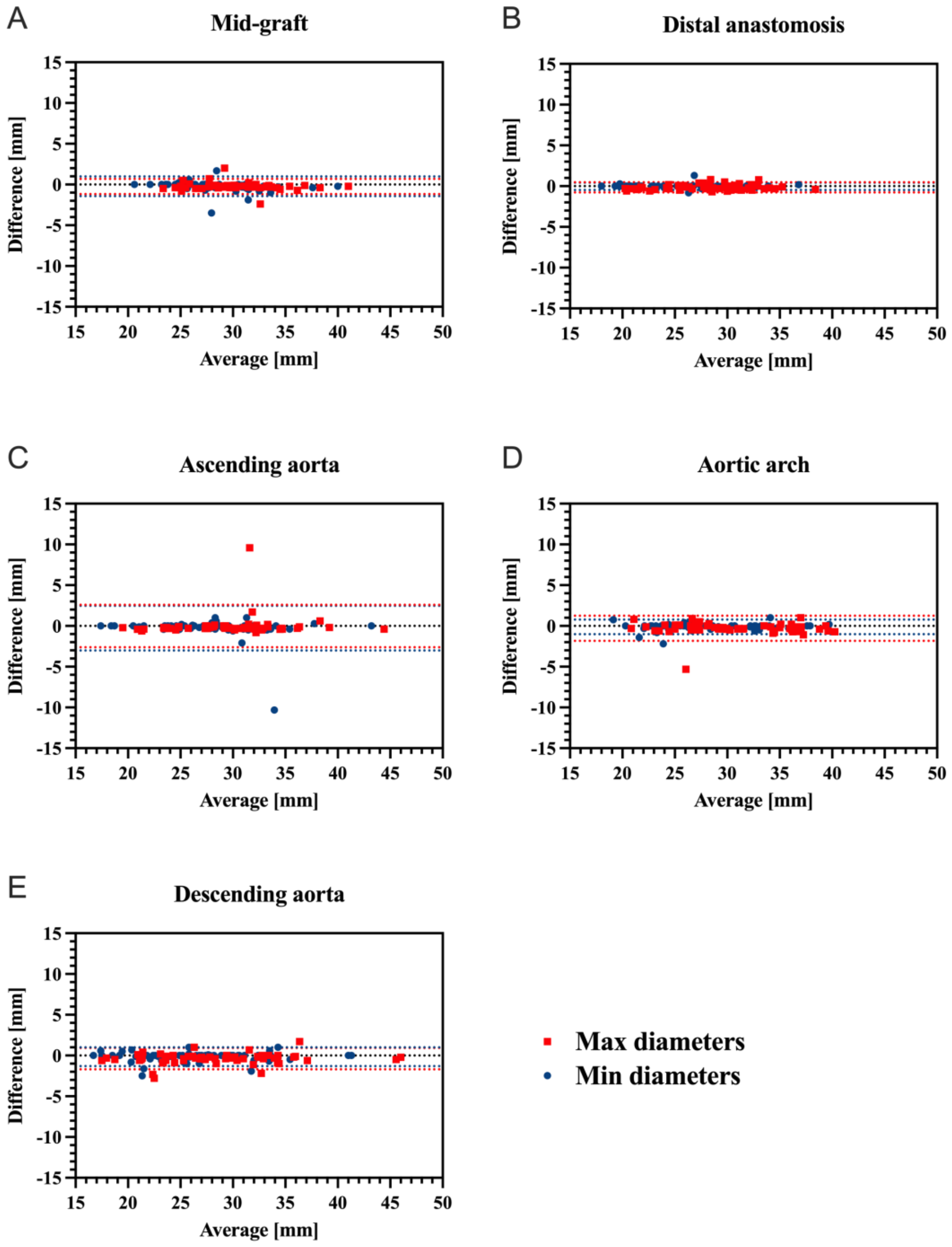

Suppl. figure 3: Bland-Altman plots for interobserver agreement between reader 1 and reader 2 regarding maximum (red) and minimum (blue) aortic diameters at the different aortic levels in REACT (mid-graft (A), distal anastomosis (B), ascending aorta (C), aortic arch (D), and descending aorta (E). Upper and lower red/blue dotted lines indicate the corresponding 95% limits of agreement. REACT=Relaxation-Enhanced Angiography without Contrast and Triggering.

Interobserver agreement CE-MRA

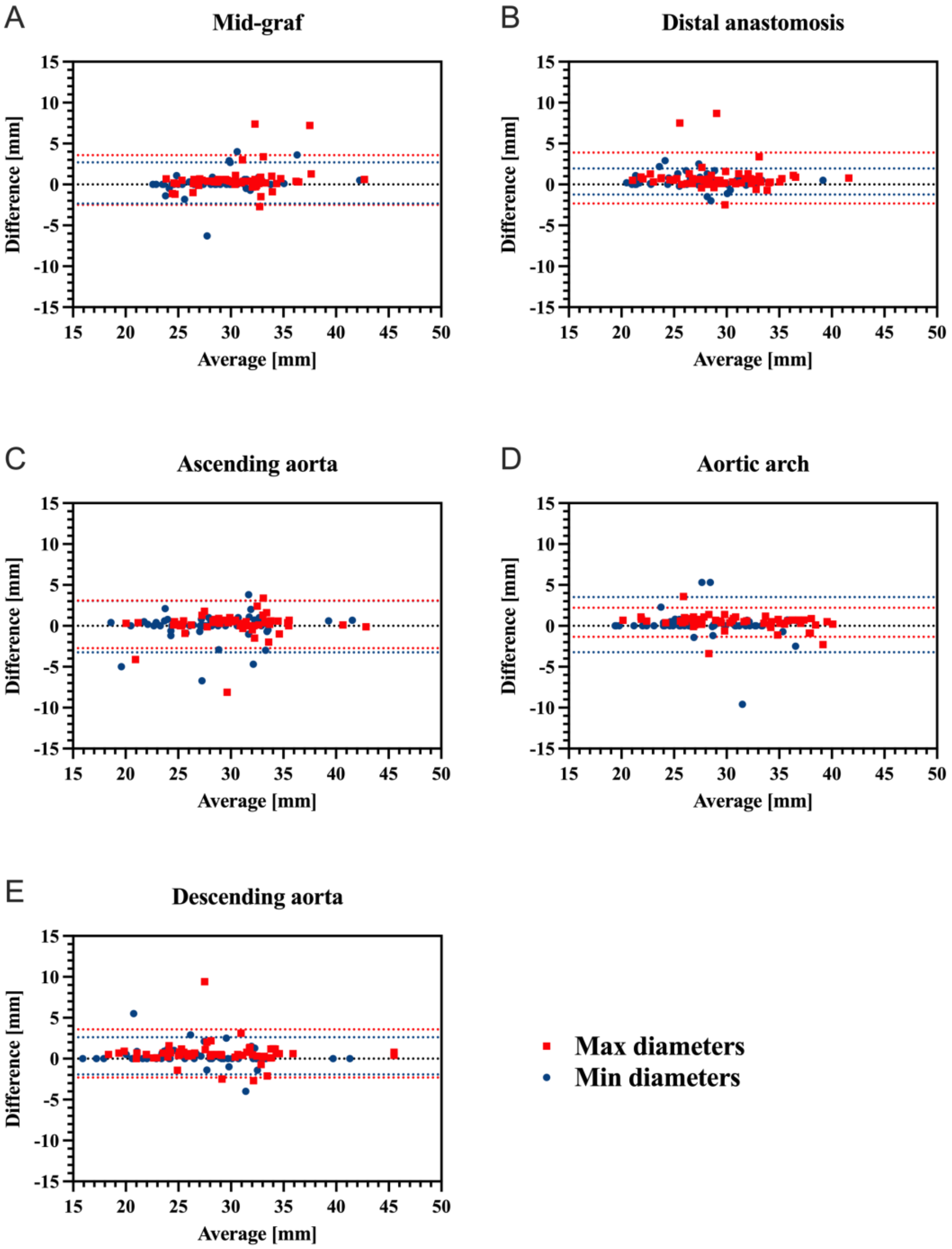

Suppl. figure 4: Bland-Altman plots for interobserver agreement between reader 1 and reader 2 regarding maximum (red) and minimum (blue) aortic diameters at the different aortic levels in CE-MRA (mid-graft (A), distal anastomosis (B), ascending aorta (C), aortic arch (D), and descending aorta (E). Upper and lower red/blue dotted lines indicate the corresponding 95% limits of agreement. CE-MRA=contrast-enhanced magnetic resonance angiography.

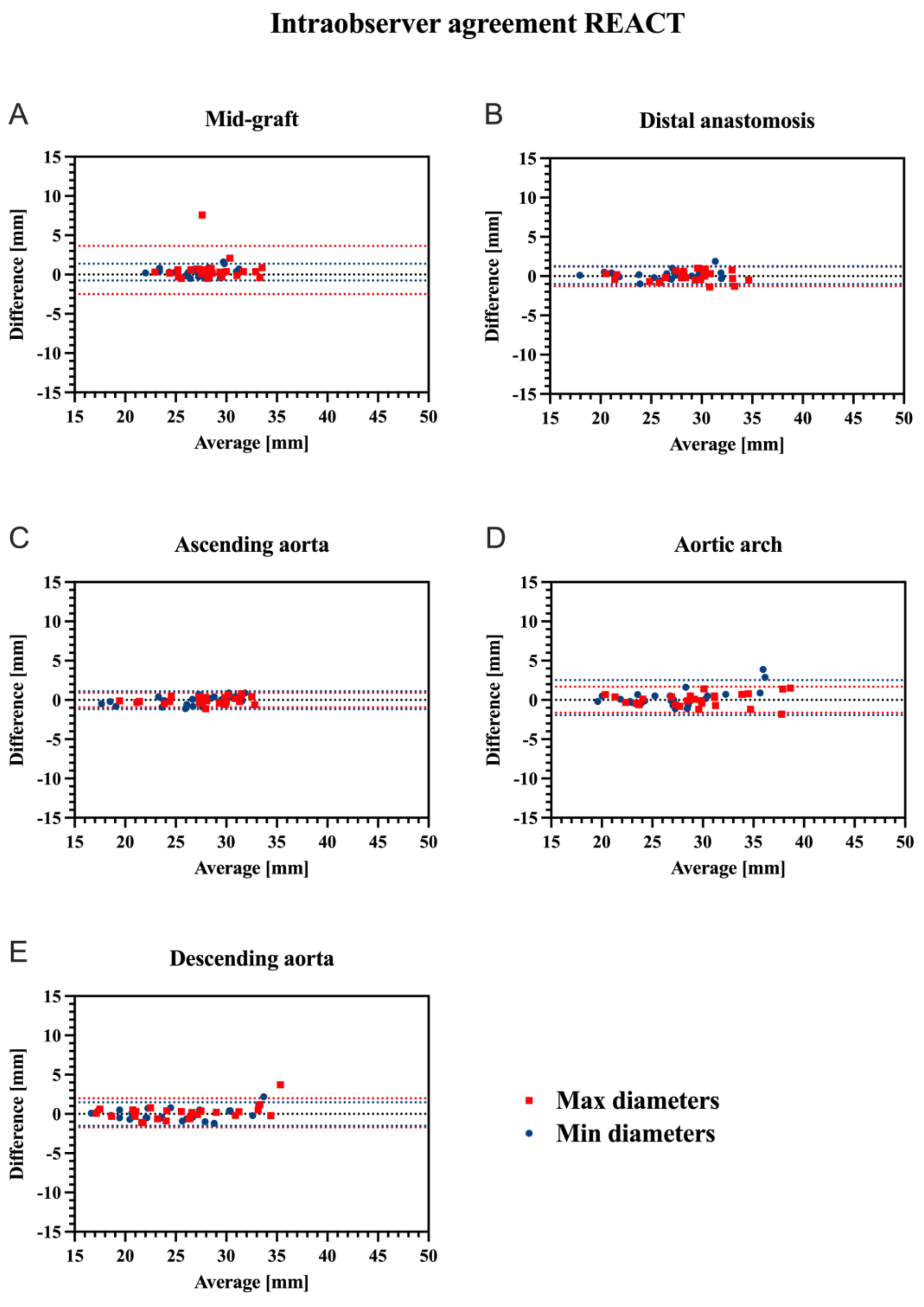

Suppl. figure 5: Bland-Altman plots for intraobserver agreement of reader 1 regarding maximum (red) and minimum (blue) aortic diameters at the different aortic levels in REACT (mid-graft (A), distal anastomosis (B), ascending aorta (C), aortic arch (D), and descending aorta (E). The second reading of 25 random cases was performed six months after the initial assessment. Upper and lower red/blue dotted lines indicate the corresponding 95% limits of agreement. REACT=Relaxation-Enhanced Angiography without Contrast and Triggering.

Intraobserver agreement CE-MRA

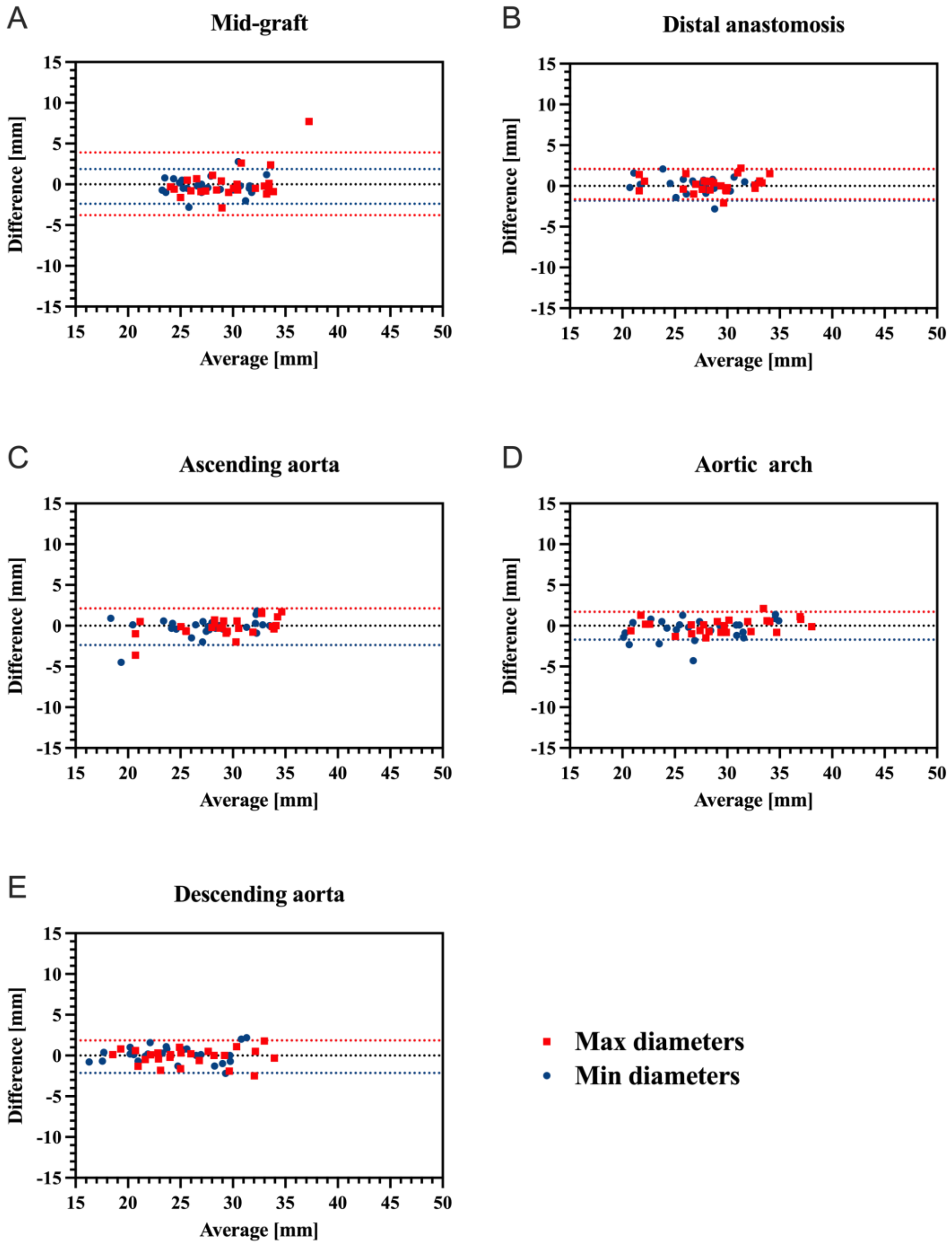

Suppl. figure 6: Bland-Altman plots for intraobserver agreement of reader 1 regarding maximum (red) and minimum (blue) aortic diameters at the different aortic levels in CE-MRA (mid-graft (A), distal anastomosis (B), ascending aorta (C), aortic arch (D), and descending aorta (E). The second reading of 25 random cases was performed six months after the initial assessment. Upper and lower red/blue dotted lines indicate the corresponding 95% limits of agreement. CE-MRA=contrast-enhanced magnetic resonance angiography.

**Intersequence comparison reader 1**			Mid-graft	Distal anastomosis	Ascending aorta	Aortic arch	Descending aorta
Maximum diameter	CE-MRA	Mean ± SD [mm]	30.94 ± 4.04	29.77 ± 4.17	30.36 ± 4.47	30.78 ± 4.97	28.98 ± 5.53
	REACT	Mean ± SD [mm]	30.06 ± 3.66	28.15 ± 4.17	29.85 ± 4.77	30.33 ± 5.16	28.20 ± 6.08
	Difference	Mean ± SD [mm]	0.29 ± 3.54	1.62 ± 2.51	0.51 ± 2.20	0.45 ± 1.73	0.78 ± 2.29
		95% Limits of Agreement [mm]	-6.65 to 7.23	-3.30 to 6.54	-3.79 to 4.81	-2.94 to 3.84	-3.72 to 5.27
	ICC (95% CI)		0.8614 (0.7765 to 0.9156)	0.8187 (0.7117 to 0.8886)	0.8872 (0.8165 to 0.9316)	0.9417 (0.9035 to 0.9651)	0.9222 (0.8719 to 0.9532)
	p (t test)		**.002**	**<.001**	.08	.05	**.01**
Minimum diameter	CE-MRA	Mean ± SD [mm]	28.99 ± 3.88	27.73 ± 3.82	28.59 ± 4.73	28.28 ± 4.64	27.18 ± 5.20
	REACT	Mean ± SD [mm]	28.60 ± 3.54	26.64 ± 4.20	28.01 ± 4.69	28.31 ± 4.80	26.74 ± 5.68
	Difference	Mean ± SD [mm]	0.87 ± 3.51	1.09 ± 2.26	0.59 ± 2.29	-0.02 ± 2.75	0.44 ± 1.91
		95% Limits of Agreement [mm]	-6.01 to 7.75	-3.34 to 5.53	-3.90 to 5.07	-5.42 to 5.37	-3.30 to 4.17
	ICC (95% CI)		0.8531 (0.7637 to 0.9103)	0.8413 (0.7457 to 0.9029)	0.8823 (0.8089 to 0.9286)	0.8328 (0.7329 to 0.8975)	0.9388 (0.8988 to 0.9633)
	p (t test)		.15	**<.001**	.06	.95	.09

Suppl. table 1: Maximum and minimum aortic diameters measured by reader 1 of CE-MRA compared to REACT. Bold indicates statistical significance; CE-MRA=contrast-enhanced magnetic resonance angiography; CI=confidence interval; ICC=intraclass correlation coefficient; REACT=Relaxation-Enhanced Angiography without Contrast and Triggering; SD=standard deviation.

Intersequence comparison reader 2			Mid-graft	Distal anastomosis	Ascending aorta	Aortic arch	Descending aorta
Maximum diameter	CE-MRA	Mean ± SD [mm]	30.41 ± 3.66	29.00 ± 4.28	30.18 ± 4.31	30.35 ± 5.24	28.34 ± 5.60
	REACT	Mean ± SD [mm]	30.31 ± 3.70	28.30 ± 4.12	29.87 ± 4.66	30.63 ± 5.19	28.59 ± 5.99
	Difference	Mean ± SD [mm]	0.09 ± 1.78	0.69 ± 1.79	0.31 ± 1.39	-0.28 ± 1.56	-0.24 ± 1.51
		95% Limits of Agreement [mm]	-3.39 to 3.58	-2.81 to 4.20	-2.41 to 3.04	-3.33 to 2.78	-3.21 to 2.73
	ICC (95% CI)		0.8850 (0.8131 to 0.9303)	0.9094 (0.8517 to 0.9454)	0.9520 (0.9202 to 0.9713)	0.9553 (0.9257 to 0.9733)	0.9659 (0.9430 to 0.9797)
	p (t test)		.69	**.005**	.09	.18	.23
Minimum diameter	CE-MRA	Mean ± SD [mm]	28.82 ± 3.55	27.38 ± 3.95	28.70 ± 4.54	28.14 ± 4.84	26.84 ± 5.41
	REACT	Mean ± SD [mm]	28.82 ± 3.67	26.67 ± 4.22	28.28 ± 4.95	28.43 ± 4.78	26.90 ± 5.65
	Difference	Mean ± SD [mm]	-0.01 ± 1.62	0.71 ± 1.97	0.41 ± 1.48	-0.29 ± 1.64	-0.06 ± 1.55
		95% Limits of Agreement [mm]	-3.18 to 3.16	-3.16 to 4.58	-2.49 to 3.31	-3.50 to 2.92	-3.09 to 2.98
	ICC (95% CI)		0.9012 (0.8387 to 0.9403)	0.8834 (0.8107 to 0.9293)	0.9515 (0.9194 to 0.9710)	0.9421 (0.9040 to 0.9653)	0.9614 (0.9357 to 0.9770)
	p (t test)		.97	**.008**	**.04**	.19	.78

Suppl. table 2: Maximum and minimum aortic diameters measured by reader 2 of CE-MRA compared to REACT. Bold indicates statistical significance; CE-MRA=contrast-enhanced magnetic resonance angiography; CI=confidence interval; ICC=intraclass correlation coefficient; REACT=Relaxation-Enhanced Angiography without Contrast and Triggering; SD=standard deviation.

Intraobserver agreement REACT			Mid-graft	Distal anastomosis	Ascending aorta	Aortic arch	Descending aorta
Maximum diameter	Reading 1	Mean ± SD [mm]	28.76 ± 2.83	28.56 ± 3.62	28.02 ± 3.79	29.36 ± 5.15	25.68 ± 5.50
	Reading 2	Mean ± SD [mm]	28.18 ± 2.84	28.60 ± 3.67	28.04 ± 3.66	29.34 ± 5.06	25.54 ± 5.10
	Difference	Mean ± SD [mm]	0.056 ± 1.96	0.22 ± 0.95	-0.13 ± 1.15	-0.01 ± 0.87	-0.15 ± 1.02
		95% Limits of Agreement [mm]	-3.79 to 3.90	-1.64 to 2.09	-2.38 to 2.12	-1.72 to 1.70	-2.15 to 1.85
	ICC (95% CI)		0.8477 (0.6848 to 0.9299)	0.9855 (0.9675 to 0.9936)	0.9923 (0.9827 to 0.9966)	0.9869 (0.9705 to 0.9942)	0.9844 (0.9650 to 0.9931)
	p (t test)		.08	.71	.83	.89	.47
Minimum diameter	Reading 1	Mean ± SD [mm]	27.49 ± 2.51	27.00 ± 3.83	26.78 ± 4.12	27.24 ± 4.95	24.56 ± 5.14
	Reading 2	Mean ± SD [mm]	27.18 ± 2.44	26.88 ± 3.74	26.82 ± 3.81	26.95 ± 4.29	24.58 ± 4.92
	Difference	Mean ± SD [mm]	-0.26 ± 1.09	0.12 ± 0.98	-0.21 ± 1.20	-0.45 ± 1.30	0.11 ± 1.06
		95% Limits of Agreement [mm]	-2.39 ± 1.87	-1.81 to 2.05	-2.56 to 2.14	-3.01 to 2.10	-1.96 ± 2.18
	ICC (95% CI)		0.9756 (0.9455 to 0.9892)	0.9883 (0.9736 to 0.9948)	0.9897 (0.9767 to 0.9954)	0.9698 (0.9327 to 0.9866)	0.9890 (0.9751 to 0.9951)
	p (t test)		**.01**	.30	.73	.21	.90

Suppl. table 3: Intraobserver agreement of reader 1 for maximum and minimum diameters of the aortic levels in 25 cases determined by using REACT. Reading 2 was performed six months after the initial assessment. Bold indicates statistical significance; CI=confidence interval; ICC=intraclass correlation coefficient; REACT=Relaxation-Enhanced Angiography without Contrast and Triggering; SD=standard deviation.

**Intraobserver agreement CE-MRA**			Mid-graft	Distal anastomosis	Ascending aorta	Aortic arch	Descending aorta
Maximum diameter	Reading 1	Mean ± SD [mm]	29.69 ± 4.02	28.78 ± 3.53	29.06 ± 4.49	29.49 ± 4.99	25.69 ± 4.42
	Reading 2	Mean ± SD [mm]	29.64 ± 3.15	28.56 ± 3.45	29.19 ± 3.90	29.50 ± 4.76	25.84 ± 4.42
	Difference	Mean ± SD [mm]	0.58 ± 1.56	-0.05 ± 0.63	-0.020 ± 0.47	0.02 ± 0.84	0.14 ± 0.94
		95% Limits of Agreement [mm]	-2.49 to 3.64	-1.28 to 1.19	-0.94 to 0.90	-1.63 to 1.68	-1.71 to 1.99
	ICC (95% CI)		0.8576 (0.7037 to 0.9347)	0.9628 (0.9173 to 0.9831)	0.9638 (0.9196 to 0.9839)	0.9847 (0.9655 to 0.9932)	0.9739 (0.9418 to 0.9884)
	p (t test)		.89	.25	.58	.96	.47
Minimum diameter	Reading 1	Mean ± SD [mm]	27.76 ± 3.23	27.40 ± 3.20	27.28 ± 4.43	26.88 ± 4.78	24.33 ± 4.43
	Reading 2	Mean ± SD [mm]	28.03 ± 3.19	27.28 ± 3.36	27.50 ± 4.04	27.33 ± 4.42	24.22 ± 4.38
	Difference	Mean ± SD [mm]	0.31 ± 0.55	0.12 ± 0.58	-0.04 ± 0.58	0.29 ± 1.14	-0.02 ± 0.76
		95% Limits of Agreement [mm]	-0.76 to 1.38	-1.01 to 1.26	-1.18 to 1.10	-1.94 to 2.52	-1.51 to 1.47
	ICC (95% CI)		0.9427 (0.8745 to 0.9743)	0.9563 (0.9035 to 0.9805)	0.9604 (0.9124 to 0.9824)	0.9600 (0.9114 to 0.9822)	0.9721 (0.9378 to 0.9876)
	p (t test)		.24	.55	.39	.10	.61

Suppl. table 4: Intraobserver agreement of reader 1 for maximum and minimum diameters of the aortic levels in 25 cases determined by using CE-MRA. Reading 2 was performed six months after the initial assessment. Bold indicates statistical significance; CI=confidence interval; ICC=intraclass correlation coefficient; REACT=Relaxation-Enhanced Angiography without Contrast and Triggering; SD=standard deviation.
